# Mental Healthcare Needs and Experiences of LGBT+ Individuals in Malaysia: Utility, Enablers, and Barriers

**DOI:** 10.3390/healthcare12100998

**Published:** 2024-05-13

**Authors:** Sheau Huey Ho, Amirul Hakim Shamsudin, Jun Wei Liow, Johan Ariff Juhari, Sai Ang Ling, Kyle Tan

**Affiliations:** 1 Faculty of Arts and Social Science, Universiti Tunku Abdul Rahman, Bandar Barat, Kampar 31900, Malaysia; hshuey@utar.edu.my; 2 Department of English, Faculty of Languages and Linguistics, Universiti Malaya, Kuala Lumpur 50603, Malaysia; 3 Department of Social Work and Social Administration, The University of Hong Kong, Pok Fu Lam 999077, Hong Kong; vcjunwei@connect.hku.hk; 4 Ministry of Health, Putrajaya 62000, Malaysia; johan.a.j.research@gmail.com; 5 Faculty of Social Science and Humanities, Tunku Abdul Rahman University of Management and Technology, Setapak, Kuala Lumpur 53300, Malaysia; lingsaiang.lynda@gmail.com; 6 Faculty of Māori and Indigenous Studies, University of Waikato, Hamilton 3240, New Zealand; k.tan@waikato.ac.nz

**Keywords:** LGBT, transgender, mental healthcare, counselling, psychologist, psychiatrist, Malaysia

## Abstract

Access to mental healthcare is undoubtedly of major importance for LGBT+ people worldwide, given the high prevalence of mental health difficulties due to minority stress exposures. This study drew mixed-method survey data from the community-based KAMI Survey (*n* = 696) to examine the enablers, barriers, and unmet needs experiences of LGBT+ individuals in accessing mental healthcare services in Malaysia. First, we present findings from a series of descriptive analyses for sociodemographic differences in unmet needs for mental healthcare, barriers, and satisfaction levels with different types of mental healthcare. Next, we conducted an inductive thematic analysis of open-text comments (*n* = 273), with relevance drawn to Andersen’s Behavioural Model of Healthcare. More than a quarter (29.5%) reported an unmet need for mental healthcare, and some groups (younger, asexual or queer, or participants living in non-major cities) reported higher unmet needs. More than three-fifths (60.5%) reported not knowing where to find culturally safe mental health professionals. The thematic analysis uncovered key contextual (e.g., mental health practitioners’ stance, stigma, collaborative client-care) and individual (e.g., positive expectation of mental health services and anticipated stigma) attributes that influence healthcare experiences. Participants also identified resources that facilitate healthcare utilisation, such as affordability, availability of suitable professionals, and geographical considerations. The implications of our findings for the mental healthcare practices in Malaysia were outlined.

## 1. Introduction

In this paper, we used ‘LGBT+’ as an inclusive umbrella term to refer to individuals who identify as lesbian, gay, bisexual, transgender, queer, or any other sexual orientation or gender identity that does not conform to cisheteronormative expectations. Compared to overseas countries, particularly those in the Global North, where more advancements in LGBT+ equity have been achieved, LGBT+ people in Malaysia face distinct challenges [1,2,3] due to the criminalisation of LGBT+ identities [4] stemming from colonial-era laws and Islamic Sharia laws, state-endorsed conversion camps [5], constant exposure to negative LGBT-related messages from politicians and religious leaders [6], and a lack of consideration of culturally safe care for LGBT+ people in mental healthcare services [7,8,9]. The criminalising environment targeting LGBT+ individuals has caused prolonged stress, fear, and anxiety across LGBT+ communities, especially for the ethnic and religious majority of Malay Muslims [10,11]. It is also the fundamental reason for the limited provision of LGBT-affirming mental healthcare and the lack of state funding to support mental health equity for the LGBT+ population in Malaysia. 

Globally, LGBT+ individuals have consistently reported significant mental health disparities compared to their cisgender and heterosexual counterparts [12,13], including in Southeast Asia [14]. In Malaysia, the prevalence of mental disorders amongst the adult LGBT+ population is estimated to be over double that of the general population (80.3% vs. 29.2%) [15]. Given the heightened risks of depression, anxiety, and suicidality amongst LGBT+ individuals [15] stemming from minority stressors such as widespread stigma and discrimination [2,13], it is undoubtedly clear that this population has higher mental healthcare needs [16]. Such discriminatory experiences also occur in mental healthcare in Malaysia [9] and overseas [16], where LGBT+ people anticipate receiving culturally safe care. In Malaysia, however, there is limited research on the experiences of LGBT+ individuals utilising mental healthcare, as well as the barriers and enablers for accessing equitable and culturally safe healthcare in Malaysia (see [9] for example).

Mental healthcare services in Malaysia are primarily offered by counsellors, clinical psychologists, and psychiatrists, and their services encompass counselling, therapy, psychoeducation, psychological-based interventions, assessments and diagnosis, and psychotropic medications. Government care refers to healthcare services provided, funded, or regulated by the government within the public healthcare sector. In contrast, private care involves healthcare services offered by privately owned facilities outside the public sector, emphasising specialisation, personalised care, and amenities tailored to individual preferences, albeit at a higher cost [17]. 

### 1.1. Andersen’s Behavioural Model of Healthcare Utilisation

In international studies, a prominent trend emerges, highlighting the higher unmet mental healthcare needs amongst LGBT+ individuals [16,18,19]. Studies examining LGBT+ healthcare utilisation, experiences of care, and confidence in health professionals [16,20,21] frequently employed Andersen’s Behavioural Model of Healthcare Utilisation [22], delineating three pivotal factors collectively influencing healthcare utilisation: (1) the health service environment; (2) enabling resources; and (3) predisposing characteristics. Andersen’s model helps unpack institutionalised injustice and systemic barriers faced by LGBT+ people within mental health settings [16] and outlines the factors that LGBT+ people find helpful (and unhelpful) as recipients of mental healthcare.

In Andersen’s model [20,21,22], the healthcare service environment includes factors such as limited knowledge among mental health professionals (MHPs) to provide culturally safe care for LGBT+ service users, unhelpful experiences of care, and denial of services. In mental healthcare, unhelpful experiences can manifest in various forms, notably through microaggressions [23] such as refusal of care, incorrect pronoun usage, and being asked invasive questions about LGBT+ identity that are unrelated to the purpose of the visit [16,19,21]. Enabling resources refer to necessities that ought to be present for LGBT+ individuals to seek out mental healthcare, encompassing availability of referral pathways to specialists, finance capability, geographical accessibility of care (e.g., distance and provision of online care), and training to bridge MHPs’ knowledge gaps. Predisposing characteristics involve beliefs, anticipating discrimination; sociocultural factors such as LGBT+ stigma, provider ignorance of LGBT+ identity issues, and provider discrimination; and demographic considerations such as age, ethnicity, and insurance coverage, contributing to financial barriers.

### 1.2. Objective

However, the current Andersen model’s application and recent research on its adaptation are predominantly centred in the Global North countries [16,20,21]. The criminalising law targeting LGBT+ individuals [24] and low awareness of LGBT+ needs in healthcare training [9] underscore the imperative for a specific study on enablers and barriers for LGBT+ people in Malaysia to access mental healthcare grounded in the unique sociocultural landscape for LGBT+ Malaysians. Additionally, existing LGBT+ research in Malaysia predominantly pathologises the mental health experience of LGBT+ individuals [2].

We attempted to address these gaps and needs by presenting findings from a nationwide survey using a mixed-method research design to examine the local experiences of LGBT+ individuals accessing mental healthcare services in Malaysia. The large sample size of the survey permitted us to explore within-group analyses of differential patterns of healthcare utilisation and expand on the original scope of Andersen’s model [20,22] to uncover a more localised and nuanced perspective on mental healthcare experiences for LGBT+ individuals in Malaysia. In contrast to these local studies that reinforce harmful stereotypes about LGBT+ identities, we model studies informed by a health equity framework in other less-stigmatising geographical regions to amplify Malaysian LGBT+ voices and needs in accessing mental healthcare access as an essential determinant of health [2]. 

Our primary objectives were twofold:

1. To assess the degree of unmet need for mental healthcare for a sample of LGBT+ individuals in Malaysia, including variations across socio-demographic groups.
2. To explore the experiences of accessing mental healthcare and service satisfaction for LGBT+ individuals in Malaysia.

## 2. Materials and Methods

The KAMI Survey, a project led by queer-identified researchers and allies with a wide range of expertise, including community psychology, counselling, psychiatry, language, and communication, employed an explanatory sequential mixed-method design. The Malay term “KAMI” translates to the collective first-person pronoun “we” and the survey was named as such to symbolise the project’s solidarity amongst and with the LGBT+ communities in Malaysia. The approach combined a quantitative component to determine barriers for participants in utilising mental healthcare services in Malaysia with a qualitative open-ended component for participants to elaborate. Developed in consultation with LGBT+ community organisations in Malaysia, the survey aimed to collect empirical data on factors affecting LGBT+ communities to assess social determinants of health. Available in both the English and Malay languages, the survey utilised a combination of purposive and snowball sampling. The survey was disseminated through social media and LGBT+ organisation networks, and participants were encouraged to share the word about the survey with their peers. Responses were gathered between 1st October and 16th December 2023, with the first 250 participants receiving a small remuneration (RM10 Touch ‘n Go e-wallet voucher) for completing the survey.

### 2.1. Participants

The survey received 757 valid responses; however, not all participants completed the whole survey due to attrition over a long survey. A total of 696 participants responded to the mental healthcare section, resulting in a completion rate of 91.9%. The sample spans an age range from 18 to 61 years old, with a mean age of 27.8 (SD = 6.97). Most participants identified as cisgender (44.7% men and 28.2% women), followed by 12.6% who identified as non-binary, 5.3% as transgender men, 4.9% as gender questioning, and 4.3% as transgender women. Slightly over half identified with a homosexual identity (40.3% gay and 14.1% lesbian), while three-tenths identified as bisexual or pansexual (31.2%). Other sexual identities included questioning or queer (7.5%), asexual (4.5%), and heterosexual (2.5%). Only 3.0% disclosed having an intersex variation. The three largest ethnic groups were Chinese (44.7%), Malay (29.7%), and Indian (13.9%). More than two-thirds primarily lived in Kuala Lumpur and Selangor (74.5%), one-in-twenty in Penang Island (5.5%), one-tenth in other West Malaysian states such as Johor and Malacca (13.5%), and the rest in East Malaysian states such as Sabah and Sarawak (6.6%). The median household income for the sample was between RM4,000 and RM5,999. A total of 273 participants further elaborated on their experiences of accessing mental healthcare through an open-text box.

### 2.2. Measures

*Closed-ended questions*. These single-item questions were adapted from overseas surveys that have validated the measures and were reviewed by academic experts in LGBT+ mental health and LGBT+ community organisations in Malaysia.

*Unmet need for mental healthcare*. We adopted a question from an Australian survey [18] that asked, “If ever, when did you last receive mental professional help (e.g., from counsellors and psychologists) for your emotional stress, substance use, or mental health-related issues?”. Responses were: “Never, because it was not needed”; “Never, but it would have been helpful”; “In the past month”; “In the past 6 months”; and “Longer than 12 months ago”. Participants who responded “Never, but it would have been helpful” were classified as having an unmet need for mental healthcare, “Never, because it was not needed” as not requiring mental healthcare, and the last three options as having needs met (see Table 1).

*Barriers to accessing mental healthcare*. Participants who reported an unmet need for mental healthcare were asked a follow-up question adapted from a New Zealand survey with transgender individuals [27]: “Thinking about the most recent time when you felt you needed this professional help for your mental health but didn’t receive it, which of the following reasons apply to you as an LGBT+ person?”. See Table 2 for the full response options that were derived from an earlier Malaysian study that interviewed LGBT+ young adults about barriers to professional mental health utilisation [9]. Participants were invited to select multiple responses that related to their experiences.

*Service use and satisfaction*. Participants who accessed mental healthcare in the last 12 months were asked, “In the last 12 months, have you received any of the following support for your emotional stress, substance use, or other mental health-related issues? Please select all that apply” to share the specific type(s) of support they utilised, including private care, government primary care clinics, government tertiary centres, LGBT+ community services, and community health services. A follow-up question, “How satisfied have you been with these mental health service providers?”, inquired about the level of satisfaction they had, on a 5-point scale (from “Extremely dissatisfied” to “Extremely satisfied”), with the specific mental healthcare they received.

*Open-text comments:* To capture the full breadth of mental healthcare experiences beyond the close-ended responses, we posed an open-ended question at the end of the section.

*Mental healthcare experience*. Participants were asked, “Is there anything else about your experiences in using mental healthcare services that you would like to share with us?”

### 2.3. Data Analysis

For quantitative analysis, we conducted descriptive analyses and chi-square goodness-of-fit tests to analyse demographic group differences using IBM SPSS Statistics v29. An alpha level of *p* < 0.05 was utilised to ascertain statistical significance for all analyses in this study.

For qualitative analysis, we adopted a phenomenological lens [28,29] to stay close to the participants’ open-ended responses on their experiences utilising Malaysia’s mental healthcare services. Authors SHH and AHS skimmed through the 273 responses before inductively coding them using the six steps of the reflexive thematic analytic approach [30,31]. These inductive codes were subsequently collapsed into initial themes based on their similarities. For example, inductive codes of “MHPs degrading client’s experiences” and “faced criticism for sensitivity” were clustered into an initial theme of “Unhelpful MHP attitudes”. Then, initial themes were regrouped based on their similarities into a higher order theme, such as regrouping “Unhelpful MHP attitudes” and “Helpful MHP attitudes” into “Quality and attitudes of MHPs”. Then, the themes were refined and renamed based on their properties and definitions. For example, instead of simply categorising the MHPs’ attitudes and qualities as helpful or unhelpful, we refined these themes with more descriptive sub-titles such as “Wholeheartedly accepting client’s experiences” and “Disrespectful and dismissive”. Author JWL, who had experience in qualitative research and clinical work with the LGBT+ community, was actively involved in supervising the coding process and peer debriefing to improve the finding’s credibility [32]. The positionality of this qualitative team consisted of MHPs (authors SHH and JWL) and a linguist (author AHS) who identified with different ethnicities, religions, and LGBT+ identifications and engaged these identities in reflexivity and discussed their views as insiders and outsiders to ensure participants’ experiences were justly reflected in the themes. For example, we discussed the differences in participants’ less helpful experiences with MHPs and compared them with the inductive voices of participants, the interpretation of a non-counsellor, the interpretation of a counsellor, and the interpretation of a supervisor and a counsellor. Then, the coding team agreed that an additional theme, “Discrimination”, was qualitatively distinct from the experiences in the “Disrespectful and dismissive” theme, deciding that it should be distinguished as it serves essential practical information for researchers and practitioners.

## 3. Results

### 3.1. Quantitative

Just under one-third of participants reported having received mental professional help in the last 12 months (32.8%), while 29.5% reported an unmet need for mental healthcare. More than one-fifth had accessed mental healthcare more than a year ago (22.3%), and 15.5% stated they had not required mental professional help. Significant demographic differences were observed for age, sexual orientation, gender, ethnicity, and residing states (see Table 1). Those reporting elevated unmet mental healthcare needs tended to be younger, identified as asexual or queer, resided in cities beyond Kuala Lumpur, Selangor, Johor, and Penang Island, or originated from East Malaysia. Chinese participants were less likely to report an unmet need for mental healthcare.

Table 2 outlines barriers hindering access to mental healthcare. Three-fifths reported not knowing where to locate culturally safe mental healthcare providers. Over half encountered financial obstacles or feared their provider lacked understanding of LGBT+ needs. Slightly less than half expressed concerns about their LGBT+ identity being pathologised and seen as the cause of mental health issues.

Table 3 details the types of mental healthcare accessed by participants in the last 12 months and their corresponding satisfaction ratings. Over two-thirds had utilised services from a private MHP, with over three-fifths of this group rating the service as ‘somewhat’ or ‘extremely’ satisfactory. Approximately one-tenth had utilised mental healthcare from government primary care clinics or tertiary centres, with lower satisfaction ratings reported for these services. More than four-fifths of participants who accessed LGBT+ community services reported feeling ‘somewhat’ or ‘extremely’ satisfied with the mental healthcare provided.

### 3.2. Qualitative

The thematic findings of open-text responses delineating the mental healthcare experiences of LGBT+ participants in Malaysia are presented in Table 4*,* along with the relevant quotes. The organisation of the three themes was guided by the framework outlined in Andersen’s Behavioural Model [20,21], with each theme encompassing subthemes identified through thematic analysis.

The data analysis suggested the essence of LGBT+ participants’ mental healthcare experiences being a journey with mixed feelings, with predominantly frustration and disappointment in their encounters with dismissive and non-LGBT-affirming MHPs, and, at times, feeling “lucky” to come across helpful MHPs. This highlights personal, interpersonal, and systemic issues that foster reluctance and fear in seeking help for mental health.

Our findings revealed that the helpful mental healthcare service environment was shaped by the stance adopted by MHPs, such as wholeheartedly accepting clients’ experiences, affirming LGBT+ identities, and embracing a collaborative client-care approach that involved working alongside clients through shared decision-making and mutual respect. These practices created safe environments for participants’ authentic expression and fostered effective communication and personalised care.

In contrast, the unhelpful stance amongst MHPs was characterised by disrespect and dismissal, a lack of LGBT+ inclusivity and affirmation, and the perpetuation of stigma and discrimination, as well as practitioner-centred care, which prioritised immediate symptom reduction, lack of cultural sensitivity, and failed to recognise the unique needs, identities, and cultural backgrounds of diverse clients. Such an approach might hinder effective treatment and exacerbate disparities in mental healthcare access. For instance, a participant expressed profound disappointment in an MHP who claimed to be LGBT-affirming, only to undermine their claim by suggesting conversion therapy, which not only fostered fear and apprehension but also deterred the participant from seeking further mental health support.

One participant said, “I have attended private therapy, and that counsellor was okay with administering conversion [practices]. I was young, about 15, so I couldn’t understand the weight of their words when they said that, but looking back now, that felt like whiplash. I had just come out to the therapist and they talked about how they were okay with supporting LGBT+ people AND converting them [to be cisheterosexual] if their parents wished for it to happen. Safe to say, I didn’t go back, nor have I gone for any mental health services, because I’m anxious they will respond the same way” (Nonbinary, Lesbian, Malay, age 18–19).

The environment where LGBT+ individuals felt unheard and invalidated exacerbated existing challenges in accessing mental healthcare, resulting in feelings of mistrust and reluctance to seek help. The effect can be seen in the following longer excerpt from a participant who shared the positive impact a wholeheartedly accepting and compassionate therapist had on her improved mental health. The positive experience stood in contrast with a subsequent unhelpful experience she had with a therapist that signalled mistrust and non-inclusivity through stereotyping her as living in a sinful relationship.

A participant mentioned, “When I first started therapy, I was with a good therapist [a counselling trainee at University A] who really got to the bottom of my issues and was wonderfully compassionate and understanding … He is the reason I am much better mentally … One other experience was with a [counselling] trainee [at University B] … In the introduction session, when I mentioned I was staying with my partner … she made a joke, ‘Oh, so you’re living in sin’, which affects me as I do have my experiences with religion and that makes me uncomfortable” (Cis woman, Lesbian, Chinese, age 25–29).

Enabling resources for mental healthcare in Malaysia presented various challenges. Affordability was a major barrier, with high costs of therapy, medication, and professional support limiting access to mental healthcare. Waiting times and appointments also affected the quality of care, with limited availability and lengthy waits for treatment. Inconsistent providers of mental healthcare could cause dissatisfaction due to frequent changes in MHPs and medications. Information and resources on LGBT-affirming care were scarce, with limited public details and a lack of interactive support on social media. Further, there were limited culturally safe MHPs who cared for LGBT+ individuals. Geographical disparities also emerged, with regional limitations hindering access to mental health support, especially in smaller cities and towns. In the following excerpt, a participant shared her frustration with inconsistent psychiatrists and ever-changing medications that were disruptive to her mental health.

A participant remarked, “The healthcare is not consistent, always changing doctors [psychiatrist] at every appointment, changing medication frequently. There’s no reliability” (Cis woman, Asexual, Malay, age 30–34). 

As part of the predisposing characteristics, participants’ positive expectations of mental health services were crucial, driven by the belief that engaging MHPs could yield positive outcomes and enhance coping mechanisms. However, anticipated stigma posed formidable barriers and fears for individuals disclosing their LGBT+ identity in mental healthcare settings, leading to reluctance to seek help, avoidance of addressing gender issues, and a lack of trust in healthcare professionals. For example, a non-binary participant noticed they self-censored concerns related to their bisexuality to avoid receiving discriminatory services, preventing them from benefiting fully from the therapy services.

A participant stated, “My therapist doesn’t know I’m bisexual and sometimes I worry she’ll treat me differently if she does, so it does hinder me opening up to her when some issues are directly related to my sexuality” (Nonbinary, Bisexual/Pansexual, Malay, age 20–24).

## 4. Discussion

Drawing data from one of the largest community-based surveys on LGBT+ individuals in Malaysia, this study provides crucial empirical insights into trends on unmet mental healthcare needs and enablers and barriers to accessing mental healthcare. Our findings not only contribute to the limited body of research in Malaysia on mental healthcare needs amongst LGBT+ people but also hold relevance for other nations facing similar contexts where the legal criminalisation of LGBT+ people leads to their neglect as a priority population for mental health Interventions [33], despite elevated rates of minority stressors (e.g., discrimination, family rejection, and efforts to change their sexual orientation and gender expression) that affect this population [2,5,9]. Additionally, while a similar study [9] also captured the lived experiences of the LGBT+ mental healthcare seekers, our study is novel in that it (1) gathered many more participants and data focusing on finding prevalence rather than existence, (2) employed mixed-method analysis to generate a broader and deeper insight of this experience, and (3) unlike the former paper’s [9] focus on the overview of themes surrounding mental healthcare experience, our study captured the structurality of the themes, juxtaposing these with the institutional focus and macro-level barriers and facilitators that aligns with a sociological approach.

The results from our analysis demonstrated that our participants were more likely to report negative experiences in Malaysia’s mental healthcare system. More than a quarter (29.5%) of our participants expressed an unmet need for mental healthcare during a time when they were desperate to access such care; a statistic that is about six times higher than an Australian sample of trans and non-binary adults [18]. This high prevalence of unmet needs for mental healthcare demonstrates considerable room for improvement in dismantling the barriers to accessing mental health services for LGBT+ individuals in Malaysia. The finding that LGBT+ individuals were more likely to report negative experiences in mental health settings is consistent with international [18] and previous Malaysian research [9]. These inequities in mental healthcare experiences were further elucidated by three factors that we categorised based on Andersen’s Behavioural Model of Healthcare [20,21,22].

***Mental Healthcare Service Environment.*** Delving into the lived experiences of the participants in this study, their unmet mental health needs revolved around the unhelpful attitudes and approaches of MHPs. Participants reported that MHPs who were disrespectful and dismissive, non-LGBT-inclusive or -affirming, holding stigma and discrimination, and adopting a practitioner-centred approach, could impede them from disclosing their LGBT+ identity or mental health concerns to MHPs due to fear of potential repercussions such as job loss or discrimination. This reluctance to engage openly with MHPs reflects LGBT+ individuals’ fear and distrust in the mental healthcare system’s ability to provide non-judgmental and supportive care. As a result, LGBT+ individuals might avoid seeking help altogether or withhold critical information during sessions, thus ultimately hindering the effectiveness of treatment and exacerbating mental health challenges. An MHP who invalidates, disrespects, or discriminates against their LGBT+ clients in a supposedly safe space reproduces systemic oppressions that LGBT+ clients experience in their everyday lives, and it could reproduce a traumatic experience that the client encounters outside the healthcare environment [34,35]. Such practices not only violate fundamental ethics to do no harm and to treat clients with equality, but they also have detrimental effects on a client’s mental health [36,37]. Malaysia’s guidelines and policies significantly lag behind international standards and best practices, as they currently pathologise LGBT+ individuals, portraying them as afflicted with mental illness, abnormality, or an undesirable lifestyle [38]. The practice of pathologisation leads to a lack of acceptance, creating environments where individuals are compelled to conceal their true selves due to fear, concern, or pressure.

Our research also explored the environments where participants who were satisfied with their mental healthcare needs indicated that a crucial element in their care experience involves helpful stance from MHPs. This stance includes compassion, understanding, validating their experiences, affirming LGBT+ identities, and taking a collaborative stance in their treatment or intervention approaches. This finding is consistent with a previous Malaysian study [9], which highlighted the importance of empathy, non-judgmental attitudes, genuineness, and validation in creating a safe space for LGBT+ individuals. These attitudes and approaches are essential for all MHPs working effectively with LGBT+ clients. MHPs must earn the trust of their clients through their actions, rather than assuming it will automatically be granted based solely on their profession [39]. Each of us embodies various degrees of cis-heterosexist prejudices (along a spectrum) for living in this cis-heterosexist world, and MHPs who could actively reflect against their prejudices and validate their LGBT+ clients’ experiences of microaggression and discrimination can provide a corrective emotional experience critical to LGBT+ clients’ care. This reflective approach to one’s positionality aligns with the internationally recognised cultural safety protocol [8] that emphasises the equalising of power dynamics in a provider–patient relationship. In the context where LGBT+ identities are criminalised and risk being persecuted, mental health providers must reflect on their “privilege” stemming from their cisgender and heterosexual backgrounds, as well as their influential positions within healthcare settings [8]. These privileges can shield healthcare providers from recognising cis-heterosexist structures, leading them to continue dictating care pathways and outcomes for LGBT+ clients without prioritising an equitable approach to care.

Furthermore, MHPs who were open to receiving feedback from LGBT+ clients and involving them in the treatment and intervention process indirectly demonstrated their valuing and validating of their LGBT+ clients’ experiences in therapy. This aligns with a review of mental healthcare experiences of LGBT+ individuals [19] that reported participants sought informed care that did not pathologise their LGBT+ identity [40] or assumed that mental health symptoms were associated solely with LGBT+ identity issues [41]. The dearth of education focusing on the needs of LGBT+ clients is a primary reason for the low level of LGBT+ competence amongst healthcare practitioners in Malaysia to effectively address the needs of the LGBT+ population [9]. Our findings corroborate this trend, as participants reported the least satisfaction with mental healthcare services provided at government primary care clinics and tertiary centres. In contrast, satisfaction ratings doubled for LGBT+ community services, where providers are trained to deliver safe and affirming mental health support tailored to LGBT+ clients [42]. LGBT+ individuals have the human right to access mental healthcare that promotes the principles of equity, inclusion, and respect for diversity [19]. The three core competency attributes that are essential for working with LGBT+ clients comprise awareness (e.g., understanding the historical and current socio-political contexts contributing to the pathologisation and marginalisation of LGBT+ individuals), knowledge (e.g., comprehending mental health experiences linked to minority stress and the diverse cultural and religious understandings of LGBT+ identities), and skills (e.g., adopting an affirmative approach to sex, sexuality, and gender diversity, and seeking supervision if required) [43].

***Predisposing characteristics.*** Some of our participants reported their reluctance to seek help or address their gender and sexuality concerns due to apprehension about being stigmatised. This aligns with previous findings where participants expressed fear for their safety, particularly those aware of the societal and legal consequences of being outed or anticipating adverse reactions from professionals toward LGBT+ communities [9]. Transgender individuals exhibited distrust and decreased motivation to disclose their transgender identities when encountering general practitioners who demonstrated low levels of cultural safety regarding transgender people [21].

Conversely, some participants held the belief that engaging with mental healthcare services can yield positive outcomes and enhance their coping mechanisms. This demonstrated their acknowledgement of the need for mental healthcare. However, the fear of stigma and distrust toward MHPs might hinder some individuals from seeking help for gender and sexuality concerns and broader mental health issues. This highlights the importance of creating safe and supportive environments within mental healthcare settings, particularly for LGBT+ individuals, to encourage help-seeking behaviour and improve access to mental healthcare services [21].

***Enabling Resources.*** Our qualitative findings revealed that enabling resources such as affordable costs, timely appointment scheduling, consistent availability of suitable MHPs, access to comprehensive information and resources on LGBT+-affirming care, and nationwide coverage were inconsistently available to LGBT+ individuals across Malaysia.

The high cost of mental healthcare is a prevalent barrier for LGBT+ individuals across countries [19,20,21], including in Malaysia [9]. In Malaysia, government healthcare services are the more affordable path as the government offers services at low costs [17]. However, they encountered protracted wait times, both on-site and during the process of securing appointments for mental health services, particularly within the purview of government hospitals. Furthermore, the inconsistent provision of MHPs within government settings added to their dissatisfaction, necessitating the repetition of their mental health issues to varying MHPs. Therefore, continuity of care is essential to address these logistic barriers by establishing clear care plans, effective communication between providers, and seamless transitions between different services to enhance outcomes and provide comprehensive support to service users [44]. On the other hand, the private healthcare sector offers advantages such as personalised care and shorter waiting times but at a higher cost, which may limit access for individuals with lower income levels [17]. Our study also found that health insurance was an important factor influencing mental healthcare utilisation, echoing [45]. However, its significance in Malaysia is relatively understudied, suggesting inadequate mental healthcare infrastructure compared to Western societies. Further exploration is necessary to understand this disparity.

Our studies identified specific demographic groups with heightened rates of unmet mental health needs, particularly for younger, asexual, or queer persons residing in cities beyond Kuala Lumpur, Selangor, Johor, and Penang Island, or originating from East Malaysia. A review of barriers rural LGBT+ experience include distance from services, economic insecurity, and limited knowledge of services [19]. Also, there is little consideration at the governmental (e.g., Ministry of Health) and institutional (e.g., health professional bodies) levels to provide equitable care for this vulnerable population, despite knowing LGBT+ people are more likely than cisheterosexuals to report a perceived need for mental health services [16,19]. These findings highlight the urgent need for geo-expanded services to rural or less-developed and -populated areas and multi-level resource allocation to address regional inequalities and ensure equitable access to quality mental healthcare across Malaysia, regardless of their financial situation and locations.

Additionally, the lower usage of LGBT+ community services might indicate lower awareness of the service provided and the funding challenges faced by these groups in expanding their services nationwide. Our data highlights the significant role of non-governmental organisations (NGOs) and public health messaging in facilitating positive mental healthcare utilisation. For example, NGOs in Yogyakarta advocate for LGBT+ rights, provide legal support, promote anti-discrimination measures, and offer mental health support [46]. Additionally, as Malaysian LGBT+ may opt for self-help resources due to fear of judgment, self-help is identified as a barrier in Australia [47]. That said, further research on NGOs’ role in Malaysian LGBT+ mental healthcare and its correlation with public health messaging and mental healthcare accessibility in Malaysia is needed. The government should recognise NGOs’ role in the healthcare system and provide more financial and practical support to help equip NGOs with better knowledge and services because they are now trusted healthcare partners by LGBT+ people.

LGBT+ individuals’ previous unhelpful experiences with Malaysian mental health professionals resulted in the anticipated stigma of mental health services, and repeated corrective emotional experiences may be necessary to restore LGBT+ clients’ faith in using mental health services. To achieve this aim, leaders in mental healthcare institutions must collaborate with local LGBT+ community groups and design standardised training and culturally safe guidelines of care on the provision of mental healthcare services for LGBT+ people in Malaysia. These trainings and guidelines invite MHPs to expand beyond a mere understanding of cultural competency to improve the provision of culturally safe care. In culturally safe care, MHPs actively challenge power imbalances by learning the historical experiences of LGBT+ people and their safety needs, which may differ from cisgender and heterosexual groups. For instance, intimate relationships and friendships are viewed by LGBT+ clients as places where they feel valued and safe, and they often refer to their intimate partners or friends as “families of choice” [48] (p. 313). These relationships and friendships are essential coping mechanisms linked to a greater sense of belonging and improved psychological well-being [48].

### 4.1. Theoretical Implication

Several studies have extended Andersen’s Healthcare Behavioural Model [16,20,21], originally utilised to analyse factors predicting healthcare utilisation among general populations, to explore barriers and facilitators for equitable healthcare access for LGBT+ individuals. In this study, we contend that a focused examination of the Malaysian context, where pervasive structural stigma affects LGBT+ individuals, is essential to account for (1) our participants’ call for a supportive and non-judgmental mental healthcare environment and (2) Malaysia’s unique sociocultural landscape, which could yield findings that are relevant to the local community in Malaysia. Therefore, this study contributes to the literature in SEA contexts, which are scarce.

Despite Andersen’s behavioural model of healthcare utilisation being frequently used and adapted to different populations, we observed nuanced contextual differences when the model was applied to a high-stigma and low-resource context. Due to the structural stigma towards LGBT+ individuals in Malaysia, our participants reported experiencing lower levels of trust towards mental health professionals. This group expressed a desperate need for information to access LGBT-affirming care, thus NGOs became the lighthouse to signpost LGBT+ individuals to trusted healthcare providers in this disconnected mental healthcare system. In comparison to other countries where advancements in LGBT+ equity have been made, and where LGBT+ individuals have more readily available access to affirmative mental healthcare in public settings, NGOs play a crucial role as providers or facilitators of culturally safe mental healthcare experiences for LGBT+ Malaysians.

Responses from participants further highlighted that the demeanour of mental healthcare staff greatly influenced their service utilisation. In contexts of high stigma, delivering culturally safe care demands that healthcare practitioners actively address power imbalances, as advocated by our participants who called for prioritising collaborative client-care over a practitioner-centred approach. This transition fosters shared decision-making between practitioners and clients and resonates with the cultural safety approach [8], which warrants further exploration in Malaysia and other countries where a practitioner-centred approach is prevalent.

We also encourage future research to consider these nuanced cross-cultural differences in enhancing the model’s applicability and relevance in diverse sociocultural contexts.

### 4.2. Practical Implications

**For MHPs:** It is crucial for MHPs to undergo comprehensive training in gender and sexual diversity and cultural competence and safety to foster a welcoming and inclusive atmosphere for LGBT+ individuals. This training should include (but is not limited to) education on diverse sexual orientations and gender identities, sensitivity to the unique challenges faced by LGBT+ individuals, and strategies for creating affirming therapeutic spaces.

**For policymakers:** Policymakers should prioritise resource allocation and initiatives that promote cultural safety in healthcare environments and support LGBT-affirming training for MHPs. An equity lens should be applied to bridge mental healthcare inequities for LGBT+ communities by allocating equitable funding and opportunities for local LGBT+ community organisations to offer LGBT-affirming care and conduct research that continuously identifies gaps in the health system.

**For advocates and allies:** Individuals and organisations advocating for LGBT+ rights and healthcare equality can leverage our findings to raise awareness about the challenges faced by LGBT+ individuals in accessing mental healthcare. Collaborative efforts among NGOs, academia, and state actors are essential to disseminate and promote the adoption of LGBT-affirming mental healthcare practices through training and guidelines developed from community-academic collaborations, which fosters more inclusive and supportive mental healthcare environments.

### 4.3. Limitations and Future Research

Methodologically, the KAMI Survey utilised a sampling method that was likely to over-recruit participants with convenient access to support from LGBT+ community groups and social media. This method might have also favoured participants residing in major cities such as Selangor and Kuala Lumpur and a younger demographic. However, our online recruitment method was chosen to protect the safety of our research team and community members, as any in-person recruitment can risk us being harassed by conservative groups, particularly within states in the east side of West Malaysia.

Further, we were unable to discern the extent of representativeness of our sample’s demographic as there is no population-based study on the proportion of LGBT+ people in Malaysia. Therefore, we would advocate for governmental-level funding to support population-level data collection to generate nationally representative statistics about the health status of LGBT+ communities. However, this would require collaboration with LGBT+ community organisations to rebuild the trust of LGBT+ individuals in the government, which has chosen to turn a deaf ear to the community’s pleas.

Further, the set of questions on unmet needs for mental healthcare was designed under the assumption that LGBT+ service users who had accessed mental healthcare had not encountered any barriers to care. Future studies could explore how LGBT+ service users overcome the identified obstacles mentioned above. Moreover, as only 3% of our participants (<20) identified as having an intersex variation, we could not unpack the specific experiences of this group in this paper, cementing the need for future studies focusing on this subgroup.

While the model primarily focuses on pre-utilisation factors, our open-text responses did not distinctly delineate between pre-use and during-use experiences of mental health services in Malaysia. Some responses touched upon the helpfulness or unhelpfulness during services, and some did not clearly indicate if it was before or when attempting mental healthcare service, indicating a mix of pre-utilisation and utilisation experiences. Further research can capture the varying experiences in different stages of mental healthcare access.

Subsequently, our study only captured the nuances of LGBT+ people, as an overarching group, accessing healthcare in Malaysia. Further studies are required to investigate within-group differences in mental healthcare experiences including different age groups, gender groups, sexual orientations, and ethnic groups of the LGBT+ population. Concurrently, this study prepares the groundwork for the development of a more comprehensive LGBT-inclusive framework that accounts for other theoretical frameworks such as minority stress and intersectionality to address structural barriers and facilitators for accessing mental healthcare in Malaysia.

## 5. Conclusions

In conclusion, our findings of high unmet mental health needs and adverse experiences reported by LGBT+ participants demonstrate the pressing need for improved accessibility, quality, and cultural safety of mental healthcare services tailored to meet the needs of this population. Our participants overwhelmingly identified factors in the mental healthcare environment as the primary issues that influence their mental health utilisation and quality of care. These are factors also reported by LGBT+ individuals overseas [19,20,21] but are made more prominent within our sample, calling for the addressing of systemic barriers within the mental healthcare service environment, including discriminatory attitudes amongst mental health professionals and inadequate training and education on LGBT+ concerns. Additionally, our study reveals the persistent inadequacy and inconsistency of enabling resources such as affordability and accessibility for mental health services nationwide. LGBT+ individuals are not a homogeneous population, as we found that younger individuals, as well as those identifying as asexual or queer, or residing in non-major cities, reported higher levels of unmet needs for mental healthcare.

## Figures and Tables

**Table 1:** Prevalence of unmet need for mental healthcare amongst LGBT+ people in Malaysia.

	Needs Met ^a^; *n* (%)	Needs Unmet ^b^; *n* (%)	Do Not Require Mental Healthcare; *n* (%)	Chi-Square Statistics
**Age groups**				χ^2^ (6) = 28.84, *p* < 0.001
18–24	145 (54.7)	**91 (34.3)**	29 (10.9)	
25–34	191 (58.8)	86 (26.5)	48 (14.8)	
35–44	41 (49.4)	21 (25.3)	**21 (25.3)**	
45+	**6 (26.1)**	7 (30.4)	**10 (43.5)**	
**Sexual orientation**				χ^2^ (6) = 38.71, *p* < 0.001
Gay and lesbian	**184 (48.8)**	111 (29.4)	82 (21.8)	
Bisexual and pansexual	**139 (64.4)**	63 (29.2)	**14 (6.5)**	
Questioning	**40 (71.4)**	11 (19.6)	5 (8.9)	
Others (including asexual, queer, and heterosexual)	19 (43.2)	**20 (45.5)**	5 (11.4)	
**Gender groups**				χ^2^ (10) = 49.09, *p* < 0.001
Trans man	25 (67.6)	10 (27.0)	2 (5.4)	
Trans women	20 (66.7)	8 (26.7)	2 (6.7)	
Non-binary	**63 (71.6)**	21 (23.9)	**4 (4.5)**	
Cis man	**134 (43.1)**	101 (32.5)	**76 (24.4)**	
Cis woman	**122 (62.2)**	54 (27.6)	**20 (19.2)**	
Questioning	19 (55.9)	11 (32.4)	4 (11.8)	
**Ethnic groups ^c^**				χ^2^ (8) = 20.81, *p* = 0.008
Malay (includes Indigenous peoples in the Malay Peninsula)	118 (56.7)	68 (32.7)	22 (10.6)	
Chinese	166 (53.4)	**79 (25.4)**	66 (3.7)	
Indian	52 (53.6)	33 (34.0)	12 (12.4)	
Bumiputera of Sabah and Sarawak	22 (51.2)	18 (41.9)	3 (7.0)	
Others (e.g., Punjabi and other Southeast Asian)	25 (67.6)	7 (18.9)	5 (13.5)	
**States**				χ^2^ (8) = 21.04, *p* = 0.007
Kuala Lumpur and Selangor	**278 (58.8)**	**123 (26.0)**	72 (15.2)	
Johor	12 (48.0)	7 (28.0)	6 (24.0)	
Penang Island	15 (42.9)	13 (37.1)	7 (20.0)	
Other West Malaysian states	29 (48.3)	**25 (41.7)**	6 (10.0)	
East Malaysian states	19 (45.2)	**21 (50.0)**	2 (4.8)	

^a^ This group includes all participants who had accessed mental healthcare at least once in their lifetime. ^b^ This group includes participants who responded that they had never accessed mental healthcare, but that it would be helpful. ^c^ The categorisation of ethnic groups followed the guidelines of the Malaysian National Health and Morbidity Survey [25]. Note: Bolded cells signify adjusted residuals exceeding ±1.96, indicating a significantly larger number of cases in those cells than expected if the null hypothesis is true [26].

**Table 2:** Barriers to accessing mental healthcare as an LGBT+ person.

	*n* (%)
Did not know where to find a mental health professional who cares for LGBT+ people in a safe way	124 (60.5)
Mental healthcare costs too much	116 (56.6)
Afraid that mental health professionals would not have enough understanding of LGBT+ people	107 (52.2)
Afraid your LGBT+ identity would be seen as a mental health issue or the cause of any mental health issue	93 (45.4)
Did not trust mental health professionals with personal information about your LGBT+ identity	86 (42.0)
Afraid that you would be asked to change your LGBT+ identity through “conversion” therapy	66 (32.2)
Afraid of being misgendered or that incorrect names would be used to refer to you	14 (28.0)
Afraid that you would be seen as “crazy” for seeking mental healthcare	52 (24.9)

**Table 3:** Type of mental health support accessed and reported satisfaction level in the last 12 months.

Mental Healthcare Provider	*n* (%)	Level of Satisfaction	*n* (%)
Private mental health professional (e.g., counsellor, psychologist, psychiatrist)	164 (71.9)	Extremely dissatisfied	3 (1.8)
		Somewhat dissatisfied	20 (12.3)
		Neither satisfied nor dissatisfied	35 (21.5)
		Somewhat satisfied	66 (40.5)
		Extremely satisfied	39 (23.9)
Government primary care clinics such as Mentari and health clinics	37 (16.2)	Extremely dissatisfied	3 (8.1)
		Somewhat dissatisfied	11 (29.7)
		Neither satisfied nor dissatisfied	5 (13.5)
		Somewhat satisfied	15 (40.5)
		Extremely satisfied	3 (8.1)
Government tertiary centres such as specialist clinics and hospital wards	24 (10.5)	Extremely dissatisfied	1 (4.5)
		Somewhat dissatisfied	5 (22.7)
		Neither satisfied nor dissatisfied	8 (36.4)
		Somewhat satisfied	8 (36.4)
		Extremely satisfied	0
LGBT+ community services (e.g., PLUHO and SEED Foundation)	24 (10.5)	Extremely dissatisfied	0
		Somewhat dissatisfied	1 (4.2)
		Neither satisfied nor dissatisfied	3 (12.5)
		Somewhat satisfied	10 (41.7)
		Extremely satisfied	10 (41.7)
Community health services (e.g., PT Foundation and community healthcare clinic)	14 (6.1)	Extremely dissatisfied	0
		Somewhat dissatisfied	4 (28.6)
		Neither satisfied nor dissatisfied	3 (21.4)
		Somewhat satisfied	5 (35.7)
		Extremely satisfied	2 (14.3)

**Table 4:** Thematic findings of mental healthcare experiences amongst LGBT+ people in Malaysia.

Anderson’s Model Framing	Subtheme		Exemplar Quotes, as Typed by Participants
**Mental Healthcare Service Environment**	**Helpful MHP Stance**	**Wholeheartedly accepting the client’s experiences** Participants encountered MHPs who were compassionate and understanding, prioritising the importance of listening and validating their experiences.	*“…I was with a good therapist who really got to the bottom of my issues and was wonderfully compassionate and understanding while still providing guidance and clarity during my downiest moments. He is the reason I am much better mentally as a person today…” (Cis woman, Lesbian, Chinese, age 25–29)* *“I think the professionals who provided the services need to be sincere. I am lucky enough to have met a psychiatrist who is very professional and sincere…” (Cis woman, Bisexual/Pansexual, Chinese, age 20–24)* *“I was surprised that most of the therapists I talk to were open to my sexuality and identity, I felt like there was actually some care and the world wasn’t hating on who I am.” (Nonbinary, Questioning, Malay, age 20–24)*
		**LGBT-affirming** Participants encountered MHPs who created a safe and inclusive environment that respects and accepts diverse sexual orientations, gender identities and sex characteristics.	*“The first thing I always ask when enquiring about any kind of therapy is if the person is LGBT+ friendly because I cannot afford to spend money on someone who might be the greatest, most experienced therapist in the country but is a complete asshole to people like me…” (Trans man, Bisexual/Pansexual, Malay, age 25–29)* *“Having mental healthcare services that are queer-affirming does wonderful things for mental health issues linked to your sexuality. The services by these providers were queer-affirming and were sensitive to the specific experiences I go through as a queer person navigating a hostile climate.” (Cis man, Gay, Malay, age 25–29)* *“I’m very glad having a psychologist that understands LGBT issues when I talk about them. My psychologist is gender-affirming and I really can open up.”* *(Cis woman, Bisexual/Pansexual, Malay, age 30–34)*
		**Collaborative client-care** Participants encountered MHPs who worked alongside them through two-way communication, shared decision-making, and mutual respect.	*“My therapist is a wonderful woman, and she has actually really helped me unpack and move through life, despite my suicidal tendencies. I always opt for private therapy or psychologist appointments. I find that they take me more seriously…” (Cis woman, Lesbian, Chinese, age 18–19)* *“…The new counsellor was so much more understanding and supportive, despite not being a part of the community. When I told her I was in a gay relationship, she came back to me in the next session having done research on how that might’ve been affecting my life and decisions and how we can overcome the trauma of my coming out experience together when I’m ready.” (Cis woman, Lesbian, Indian, age 25–29)* *“It helps me a lot, even until this day where I still do homework on what I have learnt about my mental health from my therapist, even though it’s been over a year since my last visit. I am aware of my mental health and know how to overcome some of the issues because of my therapist’s help back then.”* *(Non-binary, Asexual, Did not report ethnicity, age 25–29)*
	**Unhelpful MHP’s Stance**	**Disrespectful and dismissive** Participants encountered MHPs who failed to provide adequate support, with instances of dismissiveness, mistreatment, or focus on irrelevant issues.	*“…not taking me seriously has been a theme…” (Cis woman, Bisexual/Pansexual, Bumiputera of Sabah, age 25–29)* *“Doctor was dismissive of my experiences and was not adequately trained in sensitivity training while I was trying to get a referral from the government clinic, discouraged me from going to the sessions he gave the referral to…” (Cis man, Bisexual/Pansexual, Malay, age 20–24)* *“…the psychiatrist I spoke to was dismissive of the things I was sharing … it was not a safe space to openly disclose my gender identity/sexual orientation … I am still so traumatised from the bad interaction I had with the psychiatrist.” (Nonbinary, Bisexual/Pansexual, Chinese, age 25–29)*
		**Not LGBT-inclusive/affirming** Participants encountered MHPs who made them feel unheard and misunderstood due to a lack of localised LGBT+ cultural competency and training.	*“The services provided often feel unrelatable to me because they lack the cultural competency and sensitivity needed to address the unique experiences of me as an LGBT+ individual. Many healthcare providers lack the understanding and training necessary to navigate these complexities, so sessions always left me feeling unheard and misunderstood.” (Nonbinary, Lesbian, Chinese, age 18–19)* *“…Queer affirmative counsellors that I have talked to have this westernised framework which feels ‘off’ because of its hyper-individualism. Typical counsellors don’t account for the queer experience at all, so it feels like there’s a disconnect between them and me …” (Cis man, Gay, Malay, age 25–29)* *“…I just felt that they weren’t really hearing what I was saying … Talked about how my queer background was clearly playing a major part in my not-so-great mental health state but they just kept going around to topics that I felt was not the key route of my issues. Disappointed because it was supposed to be a queer-affirming provider.” (Cis man, Gay, Malay, age 30–34)*
		**Stigma and discrimination** Participants faced unfair treatment, prejudice, or bias, such as making insensitive jokes, perpetuating stereotypes, and dismissing or judging individuals based on their sexual orientation, gender identities, or sex characteristics.	*“…in general, our public healthcare still lacks understanding to accept those within the LGBTIQ community—much work is needed to constantly remind our healthcare professionals about gender bias and that minorities are not their own choices at their birth.” (Cis man, Gay, Chinese, age 50+)* *“I saw a university counsellor 6 yrs ago after a breakup and they totally dismissed the fact that it was the root cause of my mental state, most probably because I revealed that it was with a girl (I was already out by then). The whole experience was a waste of time and honestly made me feel worse.”* *(Cis woman, Lesbian, Chinese, age 25–29)* *“…Homophobic nurses talking behind my back, saying I chose to be gay is sinful.” (Cis man, Gay, Indian, age 25–29)*
		**Practitioner-centred care** Participants encountered MHPs who adopted a therapist-knows-it-all stance, without taking account of clients’ cultural backgrounds, and without seeing the lack of individualised care as ineffective and unhelpful.	*“I’ve found that mental healthcare services are too broad, generic, and generally ineffective—speaking from personal experience. I feel like mental healthcare services should make more effort to cater to specific individuals, not a one-size-fits-all approach.” (Cis man, Gay, Chinese, age 25–29)* *“I feel like Malaysian counsellors and psychologists (I’ve seen 3 different ones so far) are very focused on a case-by-case basis and solving the current issue that you are facing. Although I have expressed interest to delve deeper and address the root causes of my mental health, psychologists rarely go as deep into the root issues here and only focus on providing tools or methods to solve your current problems. Feels very by-the-book.” (Cis woman, Bisexual/Pansexual, Chinese, age 20–24)* *“The counselling service that I received was substandard. There was a strong focus on the ‘guilt’ that I experienced for coming out to my parents. However, the counsellor failed to link my guilt with my collectivist background, and I was left to figure that part out myself.” (Cis man, Gay, Chinese, age 30–34)*
**Enabling Resources**	**Affordability** Participants faced financial obstacles in accessing mental health services due to the high costs of therapy, medication, and other professional mental health support services.		*“I think, for my own issues, I needed someone who was more experienced (which means you need to pay more). This is because I have been relying on more affordable trainee counselling services where the effectiveness of it doesn’t seem to be long-lasting.” (Cis man, Gay, Chinese, age 25–29)* *“…I only had one session due to financial constraints so I cannot say much about the long-term effects of professional mental health support, but for what it’s worth—it helped for that moment in time.” (Cis man, Gay, Bumiputera of Sarawak, age 25–29)* *“I only have two sessions, but those sessions were really helpful to me. It was a shame that I couldn’t afford to continue.” (Trans woman, Bisexual/Pansexual, Malay, age 18–19)*
	**Waiting times and appointments** Participants experienced logistic barriers including limited availability and lengthy wait times, both at the venue and in securing appointments for mental health services in government hospitals.		*“…. Government hospital therapy is too burdened and understaffed; appointments can take up to 6 months. Most people who can’t afford to go to private clinic will just be offered medicine” (Cis man, Gay, Bumiputera of Sabah, age 20–24)* *“Just accessibility and flexibility of time for mental healthcare. I think there should be centres that open past working hours. Those ugly late-night thoughts often surface after you are not busy or occupied with work.” (Cis man, Gay, Malay, age 25–29)* *“Went to a government hospital to receive a counselling session. They only have one counsellor on standby and appointments can be up to months away … Seeing psychiatrists and taking meds alone does not help.” (Nonbinary, Lesbian, Malay, age 25–29)*
	**Inconsistent providers of mental healthcare** Participants experienced dissatisfaction and felt like they were unable to effectively improve their mental health conditions due to frequent changes in MHPs and medications.		*“… It doesn’t help that with each visit, you rarely get the same doctor/counsellor, and that the assessment of each visit is different. This made me stop going to my previous government centre.” (Nonbinary, Asexual, Chinese, age 25–29)* *“Going to a government hospital, they rotate the doctors every time I go there, so I have to say the same stuff over and over again. I feel like I’m going around in circles …” (Cis woman, Lesbian, Bumiputera of Sabah, age 20–24)* *“The healthcare is not consistent doctors are always changing at every appointment (usually I see the actual psychiatrist’s Medical Officer/House Officer) and they change medication frequently. There’s no reliability. There is no option for any sort of therapy, so I can only rely somewhat on medication to manage the symptoms instead of managing the actual mental disability and illness.” (Cis woman, Asexual, Malay, age 30–34)*
	**Information and resources on LGBT+-affirming care in Malaysia** Participants found limited public information about how to access LGBT+ community services and a lack of open information and interactive mental health support on social media.		*“We should have more open-source and interactive mental health advocates in social media.” (Cis man, Gay, Malay, age 30–34)* *“NGOs like PT Foundation, PLUHO, etc.: either they have a charge or they don’t have specific, detailed help. I don’t even know how to get to them and what they can do” (Cis man, Gay, Chinese, age 20–24)* *“There’s a dire lack of information about LGBT-affirming care in Malaysia. Most of the information is hard to find.” (Trans woman, Bisexual/Pansexual, Chinese, age 20–24)*
	**Availability of suitable MHPs** Participants encountered challenges in accessing MHPs who are LGBT+ friendly and affirming, as well as financial barriers to therapy, compounded by the understaffing and high workload of government hospitals.		*“Up until 2022, I had a hard time finding any LGBT-affirming mental health services, which made it daunting for me to seek professional help. Furthermore, I’m not able to afford much of these services. Any past instances when I had attempted didn’t end well.” (Questioning, Bisexual/Pansexual, Chinese, age 20–24)* *“I believe most mental health services are sometimes made available to gay men, or that everything is tailored for the gay male experience and not so much for lesbians and bisexuals or gender-queer folks.” (Cis woman, Bisexual/Pansexual, Indian, age 35–39)* *“I think many mental health professionals are not equipped to help me navigate my sexuality and gender … I mostly rely on community to help me navigate this area of my life, and do not talk to my therapist about it (which I am told is not great). A separate point: I go to private services because I do not trust any government or government-affiliated organisations to not be discriminatory towards LGBT+ people.” (Nonbinary, Asexual, Chinese, age 30–34)*
	**Geographical** Participants experienced regional disparities and limitations in the availability of mental health support where government MHPs were scarce in smaller cities/towns, hence impeding therapeutic progress, and there was a desire for specialised and accessible mental healthcare options in specific regions.		*“Cost, cost, cost … and in Sabah, lack of access to them.” (Trans man, Lesbian, Did not report ethnicity, age 45–49)* *“Wish there were doctors who could handle our case in clinics, with ample time and in Penang. Most NGOs are concentrated in the KL area.” (Cis man, Gay, Malay, age 25–29)* *“It really depends on where you are. In Ipoh, there was only one clinical psychologist in government and she was very busy so we couldn’t progress much … I think people in Klang valley and KL have it better … Outside of Selangor, we have close to nothing.” (Questioning, Questioning, Chinese, age 25–29)*
**Predisposing characteristics**	**Positive expectations on mental health services** Participants believed engaging with MHPs could lead to positive outcomes and improve their coping mechanisms, particularly on topics such as mental health struggles, overthinking, and experiences of discrimination.		*“It’s always good to open up about everything that’s been weighing you down. The time between you and mental healthcare service professionals is sacred between you two, so you shouldn’t be afraid to be authentically yourself when sharing your problems.” (Cis man, Gay, Bumiputera of Sabah, age 25–29)* *“Used the service that time because I just needed someone to talk to me, which was better than me overthinking.” (Nonbinary, Bisexual/Pansexual, Chinese, age 25–29)* *“That is indeed helpful to seek help and important to be recognized.” (Cis woman, Lesbian, Chinese, age 45–49)*
	**Anticipated Stigma** Participants experienced reluctance to seek help, avoidance of addressing gender and sexuality concerns, and a lack of trust in healthcare professionals due to fear of being stigmatised, thereby impeding open communication and effective treatment.		*“Trust is mutual, and it’s really hard to find LGBT+ friendly doctors. As a patient and LGBT+, I’m afraid of seeking medical services, sometimes skipping them altogether to avoid my gender issues.” (Trans woman, Bisexual/Pansexual, Chinese, age 30–34)* *“I’m scared that it will affect what I have right now with the privilege of being a normal neurotypical person on paper. I’m scared I’ll lose jobs and be discriminated if my disabilities and mental illness are made known.” (Trans man, Asexual, Chinese, age 20–24)* *“I felt like I could not speak up to my therapist at the Klinik Kerajaan (government hospital) as I was too afraid to even speak about my sexuality; thought it would’ve been a buzzkill for her, plus I felt like she wouldn’t even help at that point. (Questioning, Gay, Indian, age 20–24)*

Note. MH = mental health. MHPs = mental health professionals.

## Data Availability

The data presented in this study are available on reasonable request from the corresponding author.
